# Overexpressed cyclophilin B suppresses aldosterone-induced proximal tubular cell injury both *in vitro* and *in vivo*

**DOI:** 10.18632/oncotarget.12503

**Published:** 2016-10-06

**Authors:** Bin Wang, Lilu Lin, Haidong Wang, Honglei Guo, Yong Gu, Wei Ding

**Affiliations:** ^1^ Division of Nephrology, Huashan Hospital and Institute of Nephrology, Fudan University, Xuhui, Shanghai, P.R. China; ^2^ Division of Nephrology, The Fifth People's Hospital of Shanghai, Fudan University, Shanghai, P.R. China; ^3^ College of Animal Science and Veterinary Medicine, Shanxi Agricultural University, Taigu, Shanxi, P.R. China

**Keywords:** tubular cell, oxidative stress, endoplasmic reticulum stress, apoptosis, aldosterone, Pathology Section

## Abstract

The renin-angiotensin-aldosterone system (RAAS) is overactivated in patients with chronic kidney disease. Oxidative stress and endoplasmic reticulum stress (ERS) are two major mechanisms responsible for aldosterone-induced kidney injury. Cyclophilin (CYP) B is a chaperone protein that accelerates the rate of protein folding through its peptidyl-prolyl cis-trans isomerase (PPIase) activity. We report that overexpression of wild-type CYPB attenuated aldosterone-induced oxidative stress (evidenced by reduced production of reactive oxygen species and improved mitochondrial dysfunction), ERS (indicated by reduced expression of the ERS markers glucose-regulated protein 78 [GRP78] and C/-EBP homologous protein [CHOP]), and tubular cell apoptosis in comparison with aldosterone-induced human kidney-2 (HK-2) cells. The in vivo study also yielded similar results. Hence, CYPB performs a crucial function in protecting cells against aldosterone-induced oxidative stress, ERS, and tubular cell injury via its PPIase activity.

## INTRODUCTION

Activation of the renin-angiotensin-aldosterone system (RAAS) is a major hallmark in the development and progression of organ damage in chronic kidney disease (CKD) [1, 2]. In this regard, plasma aldosterone levels and renal expression of the mineralocorticoid receptor (MR) are elevated in patients with CKD and in CKD animal experimental models [3]. We also know that tubular cells are the main target of aldosterone through the regulation of MR, and excessive levels of aldosterone induce tubular cell injury [4, 5] and tubulointerstitial fibrosis [6], eventually leading to the progression of CKD.

Knowing the molecular mechanism by which aldosterone causes kidney cell injury will be helpful for improving our understanding of the pathophysiological role of aldosterone in the process of CKD and for exploring new therapeutic targets to cure the injury. An increasing number of studies have suggested that aldosterone induces substantial cell injury in a variety of renal cells, including proximal tubular cells, by activating oxidative stress [7, 8] and endoplasmic reticulum stress (ERS) [9]. Oxidative stress is characterized by the increased production of reactive oxygen species (ROS), resulting in mitochondrial dysfunction (MtD) and subsequent apoptosis [10]. The condition caused by ER dysfunctions, where there is aberrant protein folding in the ER lumen, is termed ERS, and we have previously indicated an important role for ROS-mediated ERS in aldosterone-induced human kidney-2 (HK-2) cell apoptosis [8]. Additionally, ERS-induced apoptosis is mainly mediated by C/-EBP homologous protein (CHOP), also referred to as GADD153 (growth arrest and DNA damage 153) [11].

Cyclophilins (CYP) are cellular binding proteins of the immunosuppressive drug cyclosporin A (CsA) [12, 13] and are constitutively expressed in most tissues [14]. CYP have peptidyl-prolyl cis-trans isomerase (PPIase) activity, which catalyzes protein folding in cells. Several classic isoforms of CYP, which include CYPA, CYPB, CYPC, and CYPD, have been identified and reside in distinctive cellular locales, apparently providing compartment-specific functions. CYPB is detected mainly in the ER lumen, playing a role in protein folding in the ER and performing vital functions to protect cells against ERS [15, 16]. However, there is currently no further information on the function of CYPB in aldosterone-induced kidney injury models.

Here, we focused on the direct effect of CYPB on aldosterone-induced oxidative stress, ERS, and tubular cell injury. Hence, the present study used *in vivo* and *in vitro* experiments to address the question of whether CYPB overexpression protects proximal tubular cells against aldosterone-induced injury, and if so, whether it is by improving oxidative stress and ERS.

## RESULTS

### Characterization of Cypb transgenic mice

**Figure 1:**
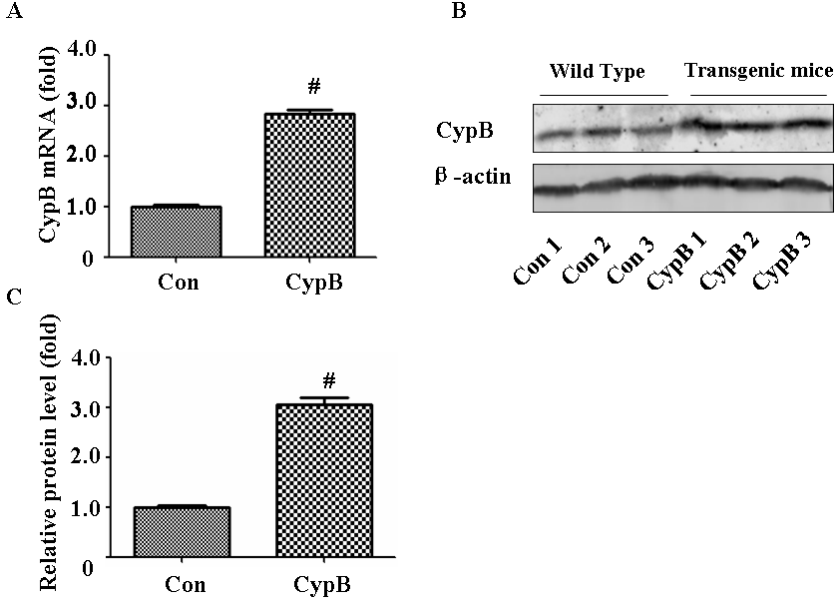
Characterization of Cypb transgenic mouse **A.** Real-time PCR analysis of *Cypb* mRNA expression normalized with *Gapdh* in wild-type and transgenic mice. **B.** Whole kidney lysate from three wild-type and transgenic mice each were immunoblotted with antibodies against CYPB and β-actin. **C.** Densitometric analysis of CYPB. Data are expressed as mean ± SEM (*n* = 3). ^#^, *P* < 0.01 *vs*. Con; Con: control/WT.

*Cypb* mRNA expression was confirmed through real-time PCR of transgenic mouse kidney (Figure 1A). The immunoreactive CYPB was also detected by western blotting in transgenic mouse kidney (Figure 1B). The expression of the *Cypb* transgene in mouse kidney was 3.1-fold greater than that from the non-transgenic littermates (Figure 1C).

### Cypb overexpression improves aldosterone-induced proximal tubular cell apoptosis *in vivo*

**Figure 2:**
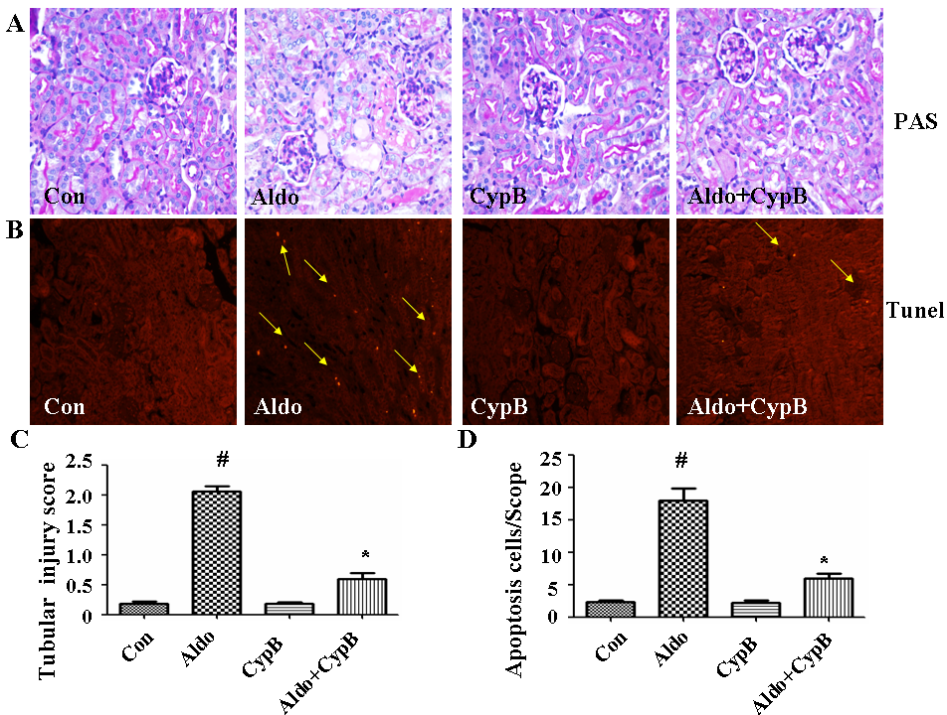
Effects of CYPB overexpression on tubular injury in aldosterone (Aldo)-induced mice **A.** Representative light microscopic appearance of PAS-stained glomeruli (×400 magnification). **B.** TUNEL assay in renal cortex sections of control and aldosterone-treated mice. Yellow arrows indicate TUNEL-positive tubular cells. **C.** Scores of tubular injury. **D.** Bar graph indicating the mean number of TUNEL-positive tubular cells per field (×200 magnification). Data are expressed as mean ± SEM (*n* = 6). ^#^*P* < 0.05 *vs*. normal control, *P < 0.05 *vs*. aldosterone alone. Con: control/WT; Aldo: Aldosterone/WT; CypB: CypB transgenic; Aldo+CypB: Aldosterone/CypB transgenic

To obtain *in vivo* data on the effect of CYPB overexpression on aldosterone-induced proximal tubular cell injury, we used a mouse model with 28-day aldosterone infusion. All physiological and biochemical data are presented in (Table 1). Aldosterone significantly increased the kidney/body weight ratio and urinary protein/creatinine ratio as compared with the control. However, *Cypb* overexpression did not affect the kidney/body weight ratio and blood pressure compared with aldosterone/salt-treated animals, but improved the urinary protein/creatinine ratio. Periodic acid Schiff (PAS) staining suggested aldosterone-induced tubular injury as indicated by the increased loss of the brush border; however, the transgenic mice only showed mild injury after aldosterone administration (Figure 2A, 2C). Further examination of renal tissues by terminal deoxyribonucleotidyl transferase-mediated dUTP-digoxigenin nick end labeling (TUNEL) assay indicated that aldosterone significantly induced tubular cell apoptosis, which was reduced in *Cypb*-overexpressing mice (Figure 2B and 2D).

**Table 1:** Biological parameters of the control, aldosterone, CYPB, and CYPB/aldosterone groups at 4 weeks

	Control	Aldosterone	CYPB	CYPB/aldosterone
Body weight (g)	26.56±1.11	26.99±1.09	25.76±0.99	25.04±1.01
Kidney/body weight ratio (mg/g)	11.99±0.24	13.51±0.34*	10.98±0.19	13.360.31^#^
Albumin/creatinine ratio (μg/mg)	39.9±3.5	121.3±23.4*	38.6±4.4	75.7±18.6**
Serum aldosterone (pg/ml)	69±5	7855±780*	71 ±6	7538±701^#^
SBP (mmHg)	110.5±3.6	163±7.6*	106±3.3	151±5.7^#^

* *P* < 0.05 *vs*. control values,

# *P* < 0.05 *vs*. CYPB,

** *P* < 0.05 *vs*. aldosterone treatment.

Control: control/WT; Aldosterone: Aldosterone/WT; CypB: CypB transgenic; CYPB/aldosterone: Aldosterone/CypB transgenic

### Cypb overexpression attenuates aldosterone-induced oxidative stress and ERS in mouse proximal tubular cells

**Figure 3:**
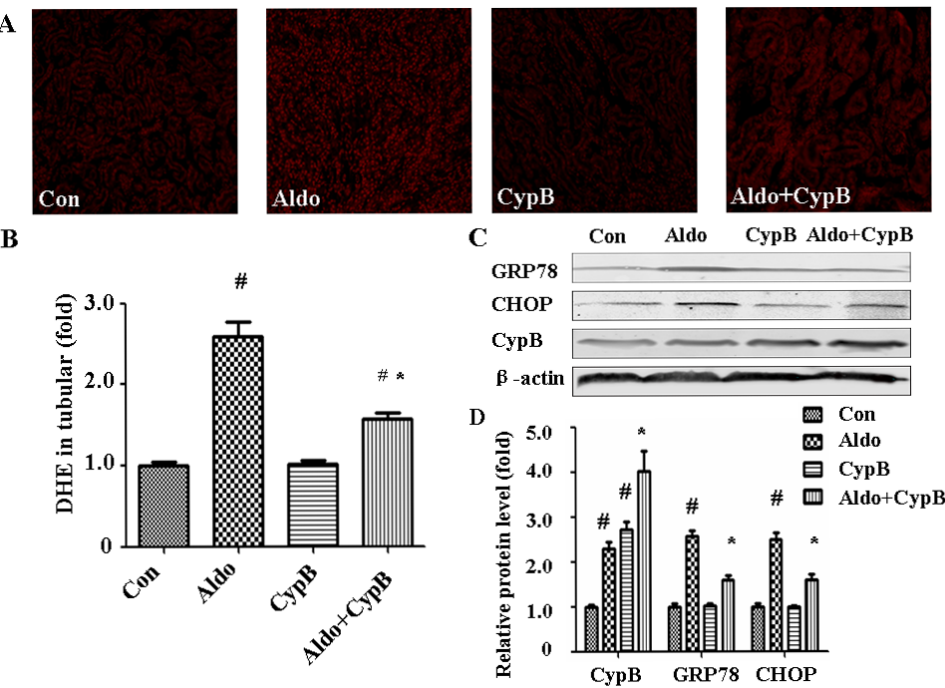
Effects of CYPB overexpression on aldosterone (Aldo)-induced oxidative stress and ERS *in vivo* **A.** DHE staining of mouse kidney sections. **B.** Bar graph indicating the mean DHE intensity per field in mouse tubular cells. **C.** Whole kidney lysate from the mice were immunoblotted with antibodies against CYPB, GRP78, CHOP, and β-actin. **D.** Graphic presentation shows the relative abundances of CYPB, GRP78, and CHOP after normalization with β-actin. Data are expressed as the mean ± SEM; *n* = 6 per group. ^#^*P* < 0.05 *vs*. control, *P < 0.05 *vs*. aldosterone group. Con: control/WT; Aldo: Aldosterone/WT; CypB: CypB transgenic; Aldo+CypB: Aldosterone/CypB transgenic

As oxidative stress is one of the major pathways contributing to aldosterone-induced kidney injury [7], we evaluated ROS levels in the tubular cells. Dihydroethidium (DHE) fluorescence in the tubular cells was markedly increased in the aldosterone group (Figure 3A and 3B). ERS is another molecular mechanism involved in aldosterone-induced tubular injury, so we evaluated the ERS markers in our animal model. As expected, the expression levels of the major ER chaperone proteins GRP78 and CHOP were increased significantly. However, these changes were improved in *Cypb*-overexpressing mice (Figure 3C and 3D). Interestingly, aldosterone infusion increased *Cypb* expression in the kidney tissue (Figure 3C and 3D).

### Aldosterone transcriptionally upregulates CYPB

**Figure 4:**
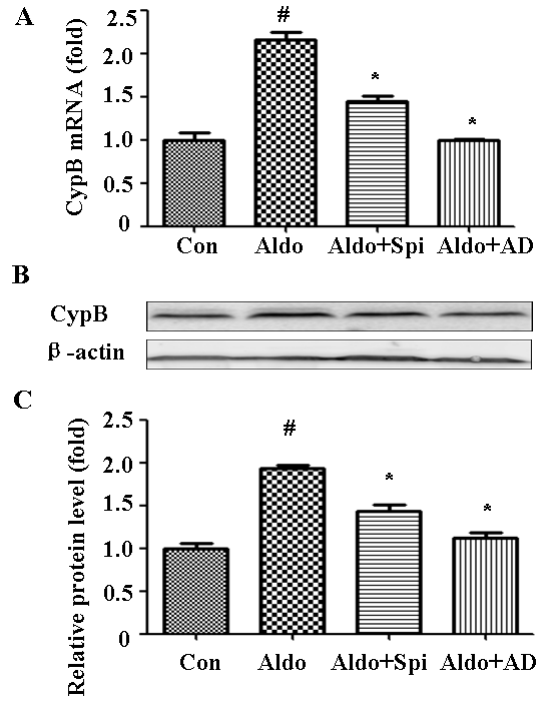
Aldosterone (Aldo) stimulation transcriptionally upregulates CYPB Equal numbers of HK-2 cells were incubated in medium containing buffer (control), Spi, or AD with or without aldosterone (10^−7^ M) for 48 h. **A.** Real-time PCR analysis of *CYPB* mRNA expression normalized with *GAPDH*. **B.** Post-treatment western blot analysis of CYPB and β-actin protein expression. **C.** Graphic presentation indicates the relative abundance of CYPB after normalization with β-actin. Results are the mean ± SEM of three experiments. ^#^*P* < 0.05 *vs*. normal control, *P < 0.05 *vs*. aldosterone alone. Con: control; Spi: Spironolactone; AD: actinomycin D

As aldosterone stimulated CYPB expression *in vivo*, we then measured whether it also occurs *in vitro*. Compared with the control cells, 48-h incubation with 10^−7^ M aldosterone significantly increased CYPB mRNA (Figure 4A) and protein expression (Figure 4B and 4C) in HK-2 cells, which suggests that aldosterone-stimulated CYPB elevation is caused by transcriptional induction. As aldosterone mediates its response through both the MR and non-genomic action, a MR antagonist (Spironolactone, Spi) and actinomycin D (AD, a transcription inhibitor) were used to characterize the role of MR in aldosterone-induced CYPB expression. Both Spi and AD abrogated aldosterone-induced CYPB transcription and expression (Figure 4A-4C), indicating that aldosterone-induced CYPB expression is dependent upon the initiation of MR-regulated transcriptional events and may serve as a self-compensative adaptive mechanism.

### CYPB overexpression suppresses aldosterone-induced apoptosis and ERS

**Figure 5:**
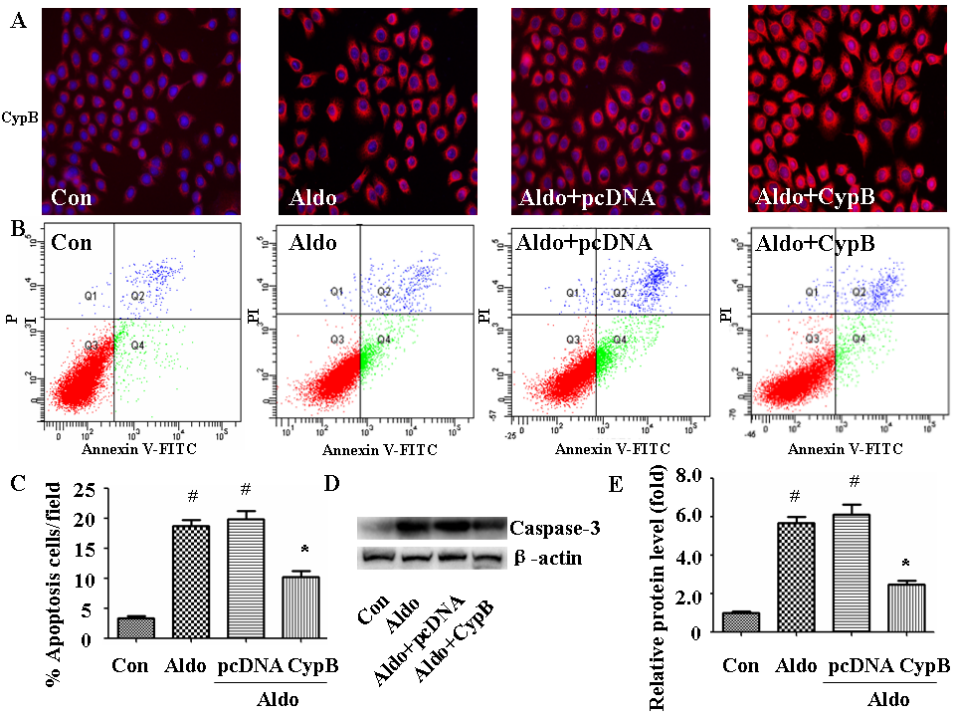
CYPB overexpression suppresses aldosterone (Aldo)-induced apoptosis **A.** Equal numbers of HK-2 cells were incubated in medium containing buffer (control), pcDNA empty vector or CYPB vector with or without aldosterone (10^−7^ M) for 24 h and CYPB immunofluorescence staining were performed. **B.** Post-treatment flow cytometry analysis of Annexin V/PI-stained HK-2 cells. **C.** Flow cytometry quantification of apoptotic cells. **D.** Western blot of Caspase-3 protein. **E.** Densitometric analysis of Caspase-3 expression. Results are the mean ± SEM of three experiments. ^#^*P* < 0.05 *vs*. normal control, *P < 0.05 *vs*. aldosterone alone. Con: control; Aldo: Aldosterone; Aldo+pcDNA: Aldosterone+ pcDNA empty vector; Aldo+ CypB: Aldosterone+ CYPB vector

**Figure 6:**
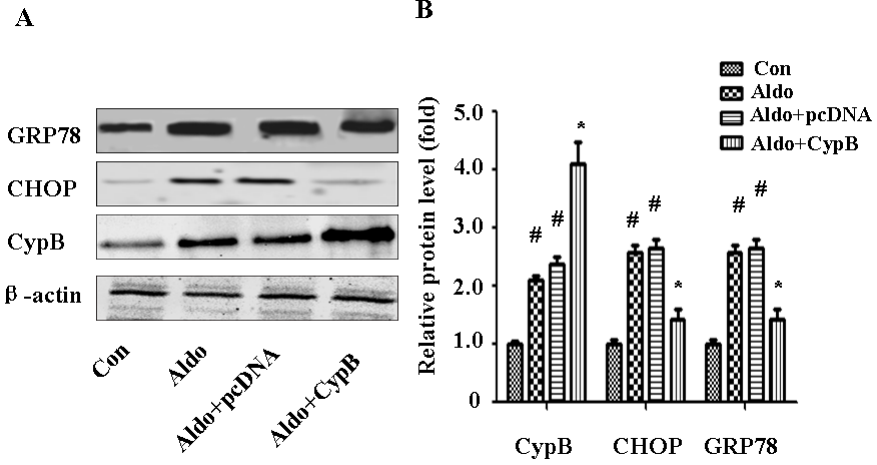
CYPB overexpression suppresses aldosterone (Aldo)-induced ERS Equal numbers of HK-2 cells were incubated in medium containing buffer (control), pcDNA empty vector or CYPB vector with or without aldosterone (10^−7^ M) for 24 h. **A.** Whole cell lysate was immunoblotted with antibodies against GRP78, CHOP, CYPB, and β-actin. **B.** Graphic presentation indicates the relative abundances of GRP78, CHOP, and CYPB after normalization with β-actin. Results are the mean ± SEM of three experiments. ^#^*P* < 0.05 *vs*. normal control, *P < 0.05 *vs*. aldosterone alone. Con: control; Aldo: Aldosterone; Aldo+pcDNA: Aldosterone+ pcDNA empty vector; Aldo+ CypB: Aldosterone+ CYPB vector

To extend our *in vivo* results to *in vitro* conditions, we used Annexin V/PI staining to evaluate the effect of *CYPB* overexpression on aldosterone-induced HK-2 cell injury (Figure 5). As expected, *CYPB* overexpression significantly attenuated aldosterone-induced apoptosis (Figure 5B and 5C). Similarly, compared with the control group, aldosterone significantly increased Caspase-3 protein. However, *CYPB* overexpression markedly decreased Caspase-3 levels (Figure 5D and 5E). Incubating HK-2 cells with 10^−7^ M aldosterone for 48 h also significantly increased the expression levels of the major ER chaperone proteins GRP78 and CHOP (Figure 6A and 6B). However, *CYPB* overexpression significantly attenuated aldosterone-induced ERS in HK-2 cells.

### CYPB siRNA induced ER stress and apoptosis in HK-2 cells

**Figure 7:**
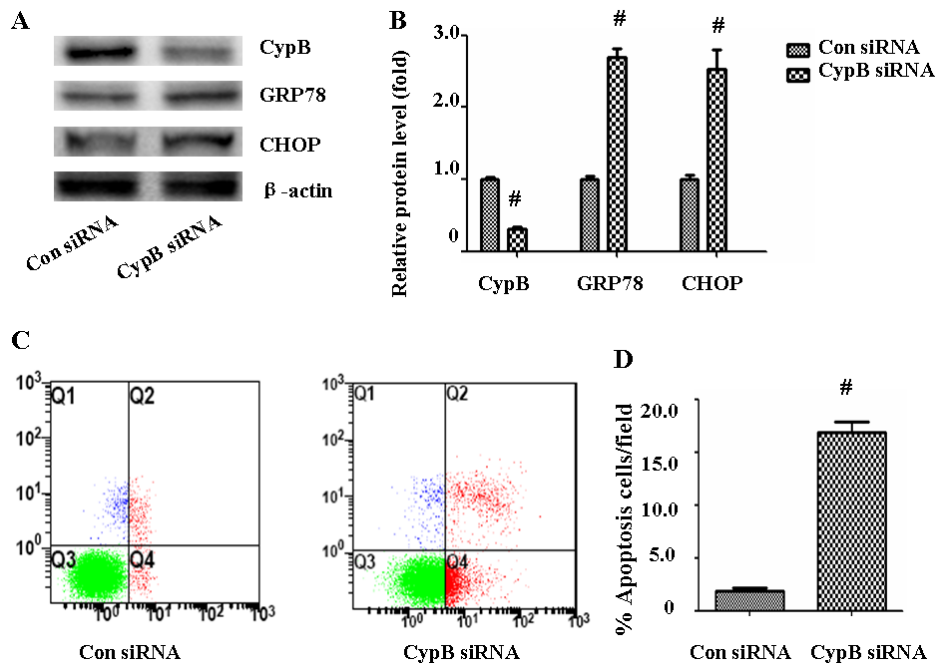
CYPB siRNA induced ER stress and apoptosis in HK-2 cells. **A.** Western blot of CYPB, GRP78, and CHOP. **B.** Densitometric analysis of CYPB, GRP78 and CHOP expression. **C.** HK-2 cell apoptosis was measured by flow cytometry. At the end of the incubation period, apoptosis was evaluated in the cells. **D.** Densitometric analysis of apoptotic HK-2 cells. Results are the mean ± SEM of three experiments. ^#^, *P* < 0.01 *vs*. Con siRNA; Con siRNA: control scramble siRNA.

We next sought to detect whether CYPB will lead to ER stress response and HK-2 cell apoptosis. As shown in Figure 7, HK-2 cells were transfected with scramble siRNA or siRNA against CYPB, and protein expression of GRP 78 and CHOP were evaluated. Treatment with 500 nM CYPB siRNA resulted in a 70% reduction of CYPB protein expression in HK-2 cells. Compared with control siRNA, CYPB siRNA significantly increased GRP78 and CHOP expression (Figure 7B). CYPB siRNA also was accompanied by HK-2 cell apoptosis (Figure 7C, 7D).

### CYPB overexpression suppresses ROS generation and mitochondrial membrane potential (MMP) depolarization

**Figure 8:**
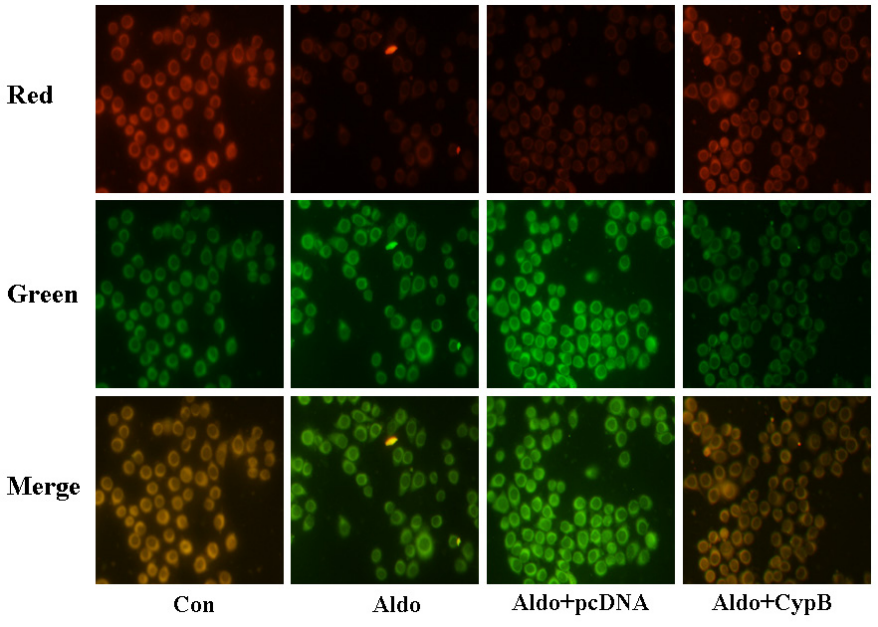
Aldosterone (Aldo) induces mitochondrial membrane potential (MMP) depolarization Equal numbers of HK-2 cells were incubated in medium containing buffer (control), pcDNA empty vector or CYPB vector with or without aldosterone (10^−7^ M) for 24 h. Representative images of HK-2 cells stained with tetrachloro-1, 1′, 3, 3′-tetraethylbenzimidazolcarbocyanine iodide (JC-1) (×400 magnification). Results are presented as the mean ± SEM (*n* = 3). Con: control; Aldo: Aldosterone; Aldo+pcDNA: Aldosterone+ pcDNA empty vector; Aldo+ CypB: Aldosterone+ CYPB vector

**Figure 9:**
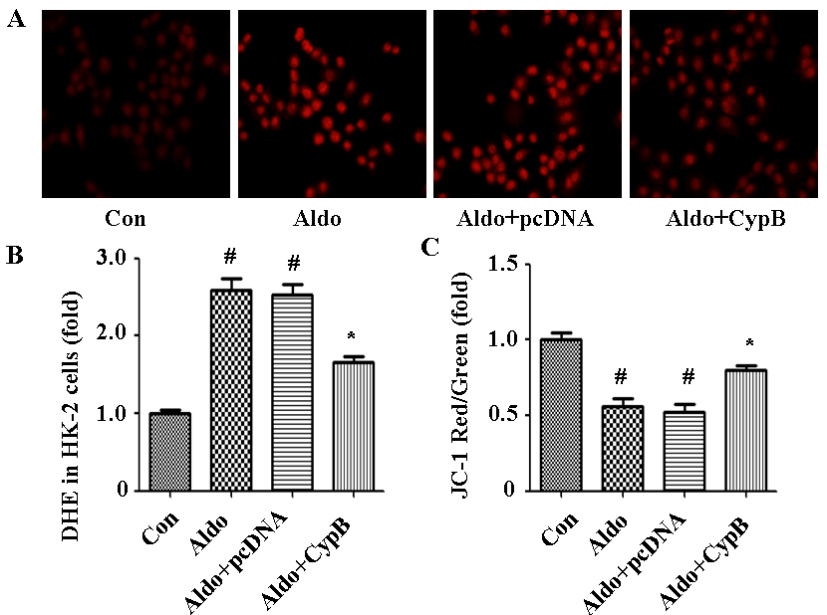
Aldosterone (Aldo) induces ROS production leading to MtD **A.** Representative images of HK-2 cells stained with DHE (×400 magnification). **B.**, **C.** DHE and JC-1 fluorescence was quantified by fluorimetry at 24 h. Results are presented as the mean ± SEM (n = 3). ^#^*P* < 0.05 *vs*. normal control, *P < 0.05 vs. aldosterone alone. Con: control; Aldo: Aldosterone; Aldo+pcDNA: Aldosterone+ pcDNA empty vector; Aldo+ CypB: Aldosterone+ CYPB vector

As it has been shown that MtD is associated with aldosterone-induced kidney cell injury [17], we hypothesized that *CYPB* overexpression may protect HK-2 cells against aldosterone-induced injury by improving MtD. We used the independent parameters ROS production and MMP to evaluate MtD. In our study, we found that aldosterone significantly increased MMP collapse (Figure 8) and DHE staining (Figure 9), and *CYPB* overexpression significantly attenuated these changes.

## DISCUSSION

The present study suggests that both oxidative stress and ERS participate in aldosterone-induced proximal tubular cell injury. However, *CYPB* overexpression attenuated all of the changes both *in vivo* and *in vitro*. Furthermore, aldosterone induced *CYPB* transcription and expression.

RAAS activation is a major hallmark in the development and progression of organ damage in CKD, and aldosterone concentrations are inappropriately high in many patients with CKD and hypertension, as well as in an increasing number of individuals with metabolic syndrome and sleep apnea [18]. Growing evidence suggests that aldosterone induces tubular injury and interstitial inflammation and fibrosis [6, 7]. Consistent with previous studies [19], our *in vivo* data suggest that aldosterone contributes to tubular cell injury, as indicated by the increased urinary protein/creatinine ratio (Table 1), loss of the brush border, and tubular cell apoptosis. Additionally, aldosterone led to cultured HK-2 cell apoptosis. These results suggest that higher serum levels of aldosterone play an important role in tubular injury in CKD. Hence, knowing the molecular mechanism by which aldosterone damages tubular cells and finding a potential intervention target is helpful for treating this condition.

The ER is one of the largest cell organelles and is responsible for post-synthesis protein modification, folding, and transport. GRP78 is a key ER regulatory protein that functions as a molecular chaperone and plays an important role in the recognition of unfolded proteins [20]. CHOP is a transcriptional factor that regulates genes involved in ERS-induced apoptosis [21]. Under normal conditions, GRP78 and CHOP are expressed at low levels. When ERS is triggered, GRP78 and CHOP are upregulated to adapt to the stress. If the stress is chronic or consistent, cells will undergo apoptosis [22]. ERS is associated with many renal disorders, including podocyte injury caused by excessive accumulation of secretory protein [23], renal ischemia-reperfusion [24], and apoptotic cell death in chronic CsA-induced nephropathy [25]. ROS produced by oxidative stress interferes with not only cellular redox-dependent reactions but also protein folding capacity, including protein disulfide bonding, ultimately resulting in protein misfolding in the ER [26, 27]. Paradoxically, ERS also increases ROS production in the ER lumen, and alteration of ER Ca^2+^ homeostasis increases cytosolic Ca^2+^, thereby stimulating mitochondrial ROS production [16]. Excessive oxidative stress is responsible for MtD, an important pathway leading to apoptosis [28]. The present study results suggest that both oxidative stress and ERS are involved in aldosterone-induced tubular damage both *in vivo* and *in vitro*.

CYPB exists in a complex with other molecular chaperones and folding enzymes [29], suggesting its possible role in protecting cells against ERS. Recent researches showed that CYPB played a protective role in various kinds of cells including macrophages, In this study, overexpression of wild-type CYPB protected tubular cells against aldosterone-induced ERS and apoptosis both *in vivo* and *in vitro*. In addition, aldosterone-treated mice had high levels of a glomerular ROS production marker. As expected, wild-type CYPB overexpression reduced the aldosterone-induced oxidative stress both *in vivo* (Figure 3) and *in vitro* (Figure 8), indicating that CYPB overexpression attenuates aldosterone-induced tubular injury by suppressing both oxidative stress and ERS. Interestingly, we demonstrate that CYPB is induced in response to aldosterone stimulation both *in vivo* and *in vitro*, suggesting a compensatory adaptive response after stress in tubular cells.

In conclusion, we provide the first evidence that CYPB suppresses excessive RAAS activation-induced oxidative stress and ERS, which may serve as a potential interventional target for improving tubular injury in patients with CKD.

## MATERIALS AND METHODS

### Ethical statement

This investigation has been conducted in accordance with the ethical standards and according to the Declaration of Helsinki and according to national and international guidelines and has been approved by the institutional review board.

### Antibodies and reagents

Aldosterone and anti-β-actin antibody were purchased from Sigma. Antibodies against CHOP, glucose-regulated protein 78 (GRP78) and Caspase-3 were purchased from Cell Signaling Technology. Anti-CYPB antibodies were obtained from Abcam. All other chemicals were of analytical grade.

### Cell culture and transient transfection of HK-2 with CYPB small interfering RNA

HK-2 cells were grown in Dulbecco's modified Eagle's medium (DMEM)/F12 containing 10% fetal bovine serum (FBS) and 1% penicillin/streptomycin (Gibco). The cells were grown at 37°C in a humidified 5% CO_2_ incubator and subcultured at 60-80% confluence using 0.05% trypsin-0.02% ethylenediaminetetraacetic acid (EDTA, Gibco). Transient transfection of HK-2 cells with siRNA was previously demonstrated [32]. HK-2 cells were grown to 60% confluence and then transfected with 500 nM CYPB siRNA or scramble siRNA (Santa Cruz, CA) using lipofectamine (Invitrogen, CA).

### Transient transfection of HK-2 cells with CYPB plasmid

A full-length human *CYPB* gene was generated by PCR and subcloned into the pcDNA3.1-HA mammalian expression vector at the *Eco*R1/*Xho*I restriction sites. The pcDNA3.1-HA empty vector was used as the control. Lipofectamine 3000 and plasmids were separately diluted in serum-free medium and incubated at room temperature for 5 min, then mixed and incubated at room temperature for a further 20 min. Aliquots of the transfection mixture were added to cell culture dishes. The medium was replaced with fresh DMEM/F12 containing 10% FBS and cultured for another 24 h after 4-h transfection. The cells were then serum-deprived for 24 h before being treated with or without aldosterone.

### Real-time PCR

Total RNA was isolated from HK-2 cells using an RNA isolation kit (Invitrogen) according to the manufacturer's instructions, and was eluted with RNase-free water. Reverse transcription was performed using a First Strand cDNA Synthesis Kit (Fermentas) according to the manufacturer's protocols. Real-time PCR was performed using THUNDERBIRD SYBR qPCR Mix reagent (TOYOBO, QPS-201) in a real-time PCR system (Stratagene). The following primer pairs were used: *CYPB* (sense 5′-GGGGACTCTGGTGTTGGAA-3′, anti-sense 5′-CGCTCCTATTGTGGCTTTGT-3′) and glyceraldehyde-3-phosphate dehydrogenase (*GAPDH*) (sense 5′-TCTTTTGCGTCGCCAGCCGAG-3′, anti-sense 5′-TCCCGTTCTCAGCCTTGACGGT-3′).

### Propidium iodide-conjugated annexin V-fluorescein isothiocyanate (FITC) staining

Annexin V is a Ca^2+^-dependent phospholipid binding protein with a high affinity for phosphatidylserine, which is externalized on the surface of the cell membrane during the progression of apoptosis. HK-2 cell apoptosis was performed according to the Annexin V/propidium iodide method (BD Biosciences, San Diego, CA) as previously described [32]. After resuspending the cell suspension evenly, one drop of suspension was placed on glass slides and the cells were observed under fluorescence microscopy; apoptosis was measured in the remaining cells using a FACScan flow cytometer (Epics Altra, Beckman Coulter).

### DHE staining

Oxidative stress was determined and quantified using microfluorimetry detection of DHE oxidation to ethidium. Superoxide (O^2^) selectively oxidizes this reaction and is an essential precursor to harmful cellular oxidants such as the hydroxyl radical (OH-) and peroxynitrite (ONOO-). Cells were cultured in black 96-well plates and the growth medium was replaced with 5 μM DHE in serum-free DMEM/F12 and incubated for 30 min. Fluorescence intensity was measured at 536 nm excitation and 610 nm emission (Synergy Mx Multi-Mode Microplate Reader; BioTek). Fluorescence values were normalized to protein content for the corresponding wells and expressed as DHE fluorescence per μg protein.

### Western blot analysis

Harvested HK-2 cells and mouse kidney tissue were lysed in sodium dodecyl sulfate (SDS) sample buffer containing 150 mM NaCl, 0.1% Triton X-100, 0.5% deoxycholate, 0.1% SDS, 50 mM Tris-HCl (pH 7.0), and 1 mM EDTA. Western blot detection of protein expression was carried out according to established protocols. The primary antibodies used were as follows: GRP78 (1:1000), CHOP (1:1000), CYPB (1:1000), Caspase-3 (1:1000) and β-actin (1:10000). Densitometric analysis was performed using Quantity One software (Bio-Rad). The relative intensity of each band was normalized to that of β-actin.

### Transgenic cypb mice

The study protocols were reviewed and approved by the Institutional Animal Care and Use Committee at Fudan University, China. PRP.ExBi-EF1α-cyclophilinB-IRES-eGFP was microinjected into the pronuclei of fertilized C57BL/6 mouse eggs that were then transferred to the oviducts of pseudopregnant foster mothers. The founder Cyclophilin B transgenic mouse was generated on a C57BL6-DBA mixed background. The mice used in this research were backcrossed six times to a C57BL6 genetic background. Genotyping was performed by PCR using the following transgene PCR primers: forward (F): 5′-AGATACACCTGCAAAGGCGGCACAA-3′ and reverse (R): 5′-GTGAACAGCTCCTCGCCCTTGCTC-3′ (for *Cypb* coding sequence), and the internal control PCR primers F: 5′-ACTCCAAGGCCACTTATCACC-3′ and R: 5′-ATTGTTACCAACTGGGACGACA-3′ (for the endogenous mouse *Actb* [β-actin] locus); tail DNA was used as the template. All experiments used mice hemizygous for the *Cypb* transgene from a single line (Cypb mice) or their non-transgenic littermates.

### Animal model

All mice were uninephrectomized and fed 1% NaCl with or without aldosterone infusion (0.75 μg/h, subcutaneously) for 4 weeks. Systolic blood pressure (SBP) was measured in conscious mice using tail-cuff plethysmography (BP-98A; Softron) at weeks 4 during the treatment period. Twenty-four hour urine samples were collected after a 24-h acclimatization period in the metabolic cages. Urinary protein excretion was determined using enzyme-linked immunosorbent assay (ELISA) kits (Exocell). Urine and plasma creatinine levels were analyzed using an assay kit (Jiancheng).

### DHE and PAS staining in kidney sections

Frozen kidney segments in OCT compound were cut into 5-μm sections and stained with DHE (50 μM, Invitrogen) for 30 min at room temperature in the dark. DHE fluorescent images were visualized under fluorescence microscopy at ×200 magnification. The average DHE fluorescence intensities of tubular cells were calculated from at least six random fields from each sample. Kidneys were also fixed with 10% formalin, embedded in paraffin, sectioned into 5 μm slices and then stained with periodic acid-Schiff (PAS). Degree of renal tubular injury was graded onto a scale from 0 to 4 as follows; 0: normal; 1: mild, involvement of less than 25%; 2: moderate, involvement of 25%-50%; 3: severe, involvement of 50%-75%; 4: extensive damage involving more than 75% of the cortex. At least 20 glomerulis were analyzed in each group [33].

### Analysis of tubular cell apoptosis in renal tissue

Apoptotic tubular cells were detected in frozen kidney sections using a FITC-labelled in situ TUNEL assay (Roche Molecular Biochemicals) according to the manufacturer's protocol. The deparaffinized sections were incubated with 50 μl TUNEL reaction mixture for 60 min at 37°C in the dark. For quantification, 10-20 fields were randomly selected from each section, and the number of TUNEL-positive cells was counted per millimeter [32].

### Statistical analyses

Data are expressed as the mean ± standard error of the mean (SEM). Comparisons between groups were performed with one-way analysis of variance (ANOVA) followed by Dunnett's multiple comparison tests or Student's *t*-test. *P* < 0.05 was considered statistically significant.
